# Transcriptomics Analysis of Wheat Tassel Response to *Tilletia laevis* Kühn, Which Causes Common Bunt of Wheat

**DOI:** 10.3389/fpls.2022.823907

**Published:** 2022-02-22

**Authors:** Ting He, Zhaoyu Ren, Ghulam Muhae-Ud-Din, Qingyun Guo, Taiguo Liu, Wanquan Chen, Li Gao

**Affiliations:** ^1^State Key Laboratory for Biology of Plant Disease and Insect Pests, Institute of Plant Protection, Chinese Academy of Agricultural Sciences, Beijing, China; ^2^Key Laboratory of Agricultural Integrated Pest Management, Qinghai University, Xining, China

**Keywords:** transcriptomic, wheat tassel, *Tilletia foetida*, defense response, wheat common bunt

## Abstract

*Tilletia laevis* Kühn [synonym *T. foetida* (Wallr.) Liro] can lead to a wheat common bunt, which is one of the most serious diseases affecting kernels, a serious reduction in grain yield, and losses can reach up to 80% in favorable environments. To understand how wheat tassels respond to *T. laevis*, based on an RNA-Seq technology, we analyzed a host transcript accumulation on healthy wheat tassels and on tassels infected by the pathogen. Our results showed that 7,767 out of 15,658 genes were upregulated and 7,891 out of 15,658 genes were downregulated in wheat tassels. Subsequent gene ontology (GO) showed that differentially expressed genes (DEGs) are predominantly involved in biological processes, cellular components, and molecular functions. Additionally, Kyoto Encyclopedia of Genes and Genomes (KEGG) enrichment analysis showed that 20 pathways were expressed significantly during the infection of wheat with *T. laevis*, while biosynthesis of amino acids, carbon metabolism, and starch and sucrose metabolism pathways were more highly expressed. Our findings also demonstrated that genes involved in defense mechanisms and myeloblastosis (MYB) transcription factor families were mostly upregulated, and the RNA-seq results were validated by quantitative real-time polymerase chain reaction (qRT-PCR). This is the first report on transcriptomics analysis of wheat tassels in response to *T. laevis*, which will contribute to understanding the interaction of *T. laevis* and wheat, and may provide higher efficiency control strategies, including developing new methods to increase the resistance of wheat crops to *T. laevis*-caused wheat common bunt.

## Introduction

Common bunt of wheat, which may have been caused by *Tilletia laevis* Kühn (synonym *T. foetida* (Wallr.) Liro), which is one of the most serious fungal diseases affecting the wheat crops globally (Goates, 2012; Bokore et al., 2019). This disease affects wheat crop growth and production *via* the infection of roots, vascular bundles of stems, leaves, tassels, and grains by replacing the grain materials and leads to a marked decline in yield and quality (Goates and Peterson, 1999; Lu et al., 2005; Goates, 2012). Common bunt can be controlled with different fungicides coated with seeds, and incorporating resistance into cultivars is still important in many wheat breeder programs worldwide and increases interest in production (Matanguihan et al., 2011; Din et al., 2021). Therefore, an extensive breeding research has been carried out to understand the genetics of disease resistance, as well as the underlying mechanisms by which new cultivars resistant to the pathogen can be produced (Dumalasová and Bartoš, 2010). Additionally, extensive and continuous use of fungicides potentially pollutes the environment and generates resistance in pathogens (Zhu et al., 2017). Therefore, an improved understanding of the defense mechanisms used by wheat crops in response to common bunt will contribute to the design of new and safer control strategies and aid in the development of resistant cultivars.

Plants have evolved in a number of strategies to effectively fight against pathogens, involving a series of morph-physiological responses, including callose deposition, cell wall modifications, hypersensitive reactions, and the production of defense-related proteins, antimicrobial metabolites, and proteins (Cohn et al., 2001; Muhae-Ud-Din et al., 2020a; Ren et al., 2020; Xu et al., 2021). These morph-physiological responses are linked with numerous pathogenesis-related genes and transcription factors. Thus, with advancement in technology, approaches in comparative “omics” have effectively donated to the effort of defining gene functions and our understanding of their expression and alterations in accumulation during plant pathogen interactions (Mathioni et al., 2011; Zhu et al., 2017; Ren et al., 2020). Transcriptome responses provide new insights into the molecular mechanisms of plant resistance during the interaction of plants and pathogens. An RNA-Seq has been evaluated for a series of host-pathogen interactions, including wheat and *T. controversa* (Ren et al., 2020), mango and *Fusarium mangiferae* (Liu et al., 2016), tomato and *Xanthomonas perforans* race T3 (Du et al., 2015), wheat and *Puccinia striiformis* f. sp. *tritici* (Poretti et al., 2021), banana and *F. oxysporum* f. sp. *cubense* (Li et al., 2013), avocado and *Rosellinia necatrix* (Zumaquero et al., 2019), wheat and *Heterodera avenae* (Kong et al., 2015), pea and *Phytophthora pisi* (Hosseini et al., 2015), and soybean and *F. oxysporum* (Lanubile et al., 2015). Based on comparative transcriptomic analysis, many mycoblastosis (MYB) transcription factors and defense-related genes (Thilmony et al., 2006; Mukherjee et al., 2010; Proietti et al., 2013) were found to be involved in defense mechanisms and in primary and secondary resistance-associated signal transduction paths of plants. For example, defense-related genes were found to be significantly upregulated in wheat after *T. controversa* inoculation (Ren et al., 2020), phytohormone-related genes were upregulated in potato after *Ralstonia solanacearum* (Zuluaga et al., 2015), and MYB transcription factor families were upregulated in wheat after *T. controversa* (Chen et al., 2021) and *R. cerealis* infections (Shan et al., 2016).

Wheat crops are staple food crops in many countries (Shewry, 2009). Although extensive research has been done to date to analyze wheat-pathogen interactions (Zhang et al., 2012; Haueisen et al., 2019; Ren et al., 2020), very little literature is known about transcriptome responses between wheat and *T. laevis*, which lead to wheat common bunt being an emerging and serious fungal disease that causes dramatic quality and quantity losses in wheat. This study is the first global transcription analysis of wheat tassels in response to *T. laevis*. to develop resistance by contributing to the control of common bunt disease.

## Materials and Methods

### Plant and Fungal Material

Healthy wheat kernels (Cultivar: Dongxuan 3) and *T. laevis* were collected from the Institute of Plant Protection, Chinese Academy of Agricultural Sciences, China. The grains were sterilized and vernalized, and ten wheat seedlings were transplanted into every pot. Based on morphological characterization of teliospores of *T. laevis* (Supplementary Figure 1) and specific band and sequence of SCAR marker of *T. laevis* (Supplementary Figure 2), we identified the pathogen of *T. laevis* based on the previous reports (Xu et al., 2020; Qin et al., 2021). Then, the identified *T. laevis* was use in this study. The teliospores of *T. laevis* were cultured following the methods of previously published reports (Muhae-Ud-Din et al., 2020a). Briefly, two to three kernels were crushed by a centrifuge machine, disinfected with 2 ml of 0.25% NaClO and cultured at 16°C and 60% relative humidity for 14 days in an incubator (LT-36VLC8, Percival, United States). After 16 days of incubation, hyphal growth was checked under an inverted microscope (IX83, Olympus, Japan). Hyphae were collected and mixed with ddH_2_O and used to inoculate wheat plants at a concentration of 10^6^ spores/ml with an OD_600_ of 0.15. One milliliter of obtained mycelium was injected into tassels, and injection was repeated for 3 times with a 1-day interval. The tassels injected with ddH_2_O were used as a control in this study. Ten pots were used for *T. laevis* inoculation and for control separately. The tassels (6 ± 5 cm in length) were detached from *T. laevis*-inoculated and control plants, with three biological replicates for every treatment.

### RNA Extraction From Wheat Tassels

Wheat tassel RNA was extracted from each sample using a total plant RNA extraction kit (Ambion, TX, United States), according to the manufacturer’s instructions. The quality and integrity of RNA were determined by using agarose gel electrophoresis, while the concentration was determined through a NanoDrop 2000 (Denovix, United States), and RNA was stored at −80°C for further use.

### Transcriptome Sequencing of Purified RNA

The raw reads were filtered by using the high-throughput sequencing Illumina HiSeq 4,000 platform. Clean reads were generated by removing reads with adaptors, reads where the number of unknown bases was more than 10%, low-quality reads (those in which more than 50% of bases presented a quality of ≤ 10) and poly-N (unrecognized bases). Clean reads were then aligned to the reference sequence^1^ using the software hisat2 (Kim et al., 2015) with the parameters set by the system.

### Kyoto Encyclopedia of Genes and Genomes and Gene Ontology Enrichment Analysis

The Kyoto Encyclopedia of Genes and Genomes (KEGG)^2^ pathway analysis was performed by using GPSeq, and gene ontology (GO) enrichment analysis was performed with FDR < 0.05, representing the significantly expressed genes (Kanehisa et al., 2008; Young et al., 2010), NR, EggNOG database, NT, KOG/COG (Tatusov et al., 2000), Pfam (Bateman et al., 2002), and SWISS-Prot (Bairoch and Apweiler, 1997).

**TABLE 1:** Transcriptome analysis of RNA-Seq data.

Sample	Raw reads	Clean reads	Total mapped reads	Uniquely mapped	Q30	GC
Control,1	49.69 M	48.59 M	42,935,380 (88.36%)	36,301,512 (74.71%)	93.75%	53.35%
Control,2	50.29 M	49.12 M	44,204,310 (89.99%)	37,732,069 (76.81%)	93.96%	52.74%
Control,3	48.64 M	47.46 M	42,391,424 (89.33%)	35,937,004 (75.73%)	93.58%	52.74%
Infected,1	51.69 M	50.69 M	24,042,553 (47.43%)	21,629,400 (42.67%)	94.63%	55.91%
Infected,2	48.75 M	47.82 M	21,748,000 (45.48%)	19,842,072 (41.49%)	94.79%	56.20%
Infected,3	51.52 M	50.30 M	40,159,626 (79.85%)	34,459,566 (68.51%)	94.50%	56.72%

*Infected-1 stands for T. laevis infected-1, Infected-2 stands for T. laevis infected-2, and Infected-3 stands for T. laevis infected-3.*

### Verification of Differentially Expressed Genes by Quantitative Real-Time PCR

First-strand cDNA was synthesized by using 1.5 μg purified RNA by using the Trans Script One-Step gDNA Removal and cDNA Synthesis SuperMix Kit (Beijing Quanshijin Biotechnology Co., Ltd., China) and stored at -20°C for further use. A PerfectStart Green qPCR SuperMix (TransGen Biotech, Beijing, China) kit was used for quantitative real-time polymerase chain reaction (qRT-PCR). The 20-μl reaction mixtures contained 1 μl template, 0.4 of each primer (10 μM), 10 μl SuperMix (Beijing Quanshijin Biotechnology Co., Ltd., China) and 8.2 μl ddH_2_O. The amplification protocol was 94°C for 30 s, followed by 40 cycles of 94°C for 5 s, 60°C for 30 s, and 72°C for 30 s. All cDNA and qRT-PCRs were carried out in triplicate. The 2^–ΔΔCt^ method was used to calculate the relative expression of each gene (Pfaffl, 2001). The primers used for validation are listed in Supplementary Table 1.

## Results

### Transcriptomic Analysis of RNA-Seq Data

**FIGURE 1:**
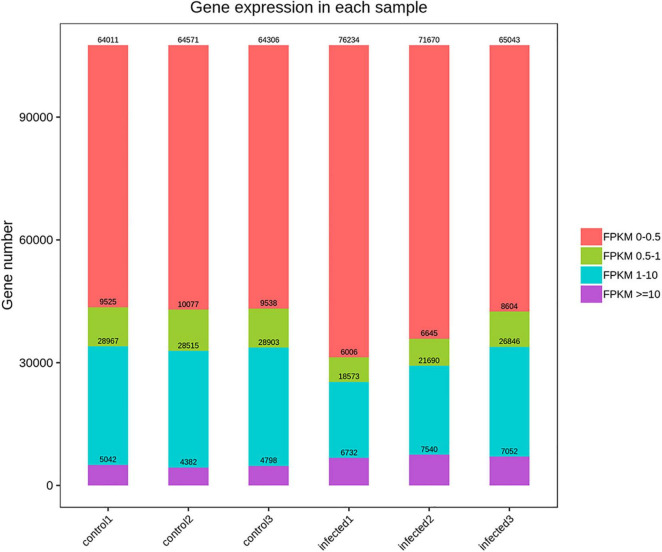
Summary of differentially expressed genes (DEGs). Numbers of DEGs between the control and *T. laevis* infection groups.

**FIGURE 2:**
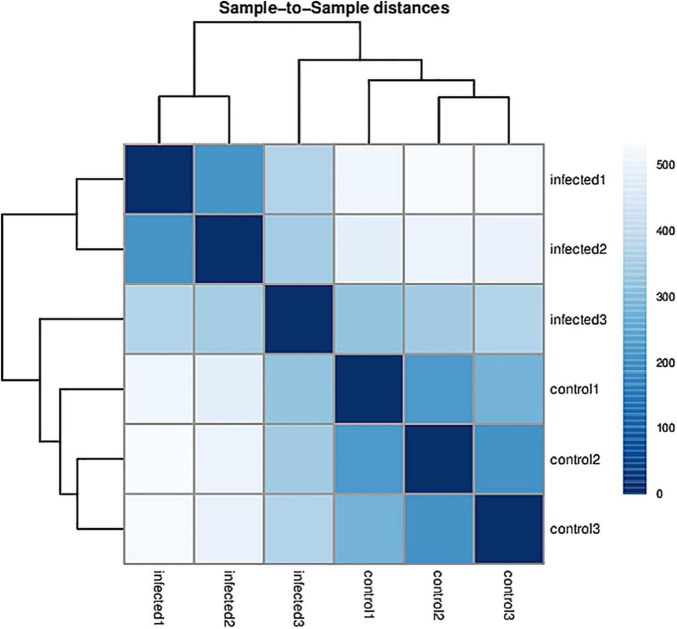
Sample-to-sample clustering analysis for checking batch effects and their similarity.

**FIGURE 3:**
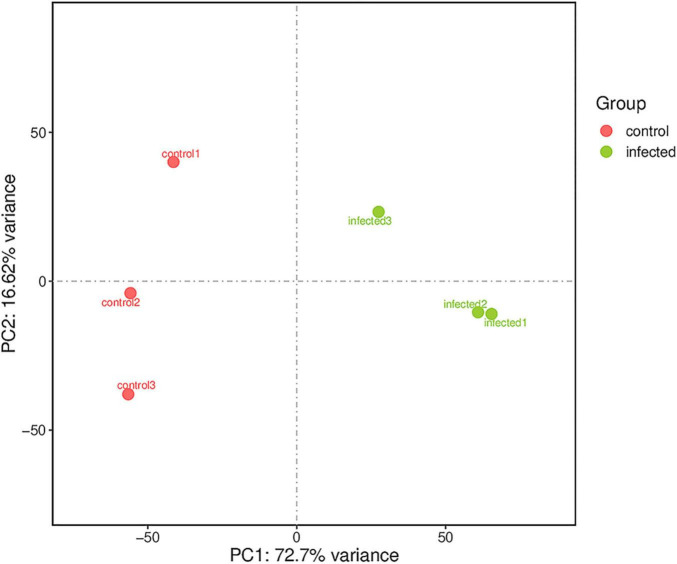
Principal component analysis (PCA) for gene expression patterns. The first and second PCAs explained 72.7 and 16.62% of the variance, respectively.

Based on RNA-Seq, we identified the alterations in wheat genes when the spike was infected by *T. laevis*. Six cDNA libraries (three *T. laevis*-infected and three control) were sequenced. Approximately 49.69, 50.29, and 48.64 million raw reads were produced from the control samples control-1, control-2, and control-3, respectively, and raw reads in *T. laevis* samples were 51.69, 48.75, and 51.52 million in infected samples, respectively. Similarly, 48.59, 49.12, and 47.46 million clean reads were produced from control samples control-1, control-2, and control-3, respectively, and clean reads in *T. laevis* samples were 50.69, 47.82, and 50.30 million in infected samples, respectively. Total mapped reads and uniquely mapped reads of the above-mentioned transcripts ranged from 45.48 to 89.99% and 41.49 to 76.81%, respectively. Additionally, the Q30 and GC of these transcripts ranged from 93.58 to 94.79% and 52.74 to 56.72%, respectively (Supplementary Table 1). Next, the differentially expressed genes (DEGs) were recognized by comparing the fragments per kilobase of transcript per million mapped reads (FPKM) value of every gene between control and *T. laevis*-infected samples. For control-1 DEGs, 5,402 (FPKM ≥ 10), 28,967 (FPKM 1–10), 9,525 (FPKM 0.5–1), and 64,011 (FPKM 0–0.5) genes were differentially expressed. For *T. laevis*-infected DEGs, 6,732 (FPKM ≥ 10), 18,573 (FPKM 1–10), 6,006 (FPKM 0.5–1), and 76,234 (FPKM 0–0.5) genes were differentially expressed. The DEGs of *T. laevis*-infected were showed (Figure 1). In addition, the sample-to-sample cluster analysis showed that the repeatability between each group of sequencing samples was high, indicating that the repeatability and reliability of the transcriptome sequencing data meet the needs of further analysis (Figure 2). Furthermore, principal component analysis (PCA) was performed for both control- and *T. laevis*-infected samples. The PCA of the samples of the infection group and the control group found that the control group was concentrated in the first and third quadrants, and the infection group was concentrated mainly in the second and fourth quadrants, indicating that there were differences and good repeatability within each group (Figure 3).

### Analysis of Differential Gene Expression Patterns

**FIGURE 4:**
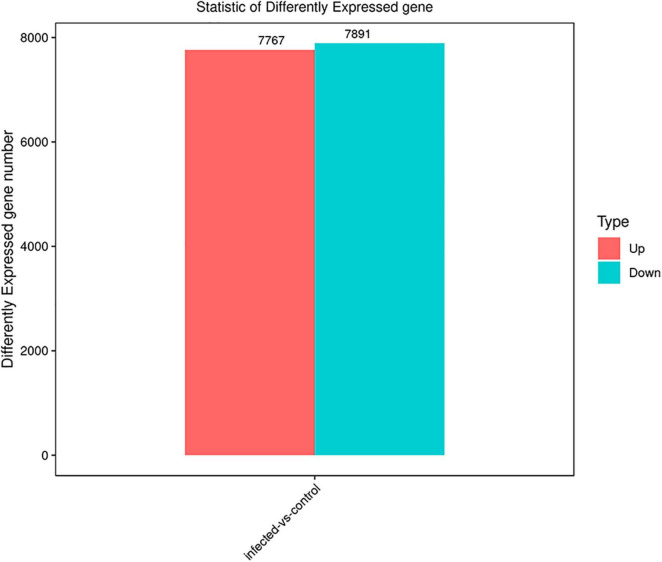
Significant differentially expressed genes (DEGs) in *T. laevis*-infected vs. control samples. Up- or downregulated DEGs in response to *T. laevis* infection.

**FIGURE 5:**
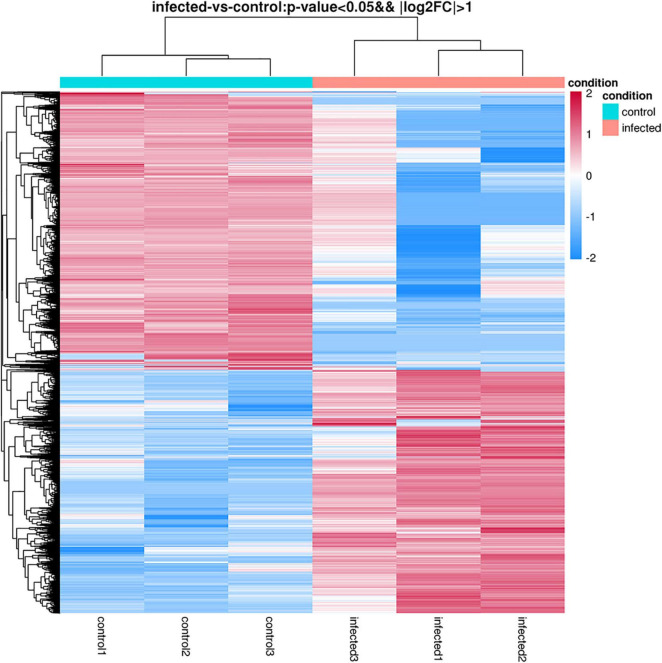
Hierarchical clustering heatmap of DEGs according to changes in expression in response to *T. laevis* infection. Each column shows a library, and each row shows a DEG expression. The colors blue, white, and red indicate low, medium, and high expression patterns of genes, respectively.

The differentially expressed genes were recognized in *T. laevis*-infected and control libraries. By comparing the tassels infected with *T. laevis* and the control, 49.6% genes (7,767 genes out of 15,658 genes) were found to be upregulated and 50.4% genes (7,891 genes out of 15,658 genes) were found to be downregulated (Figure 4 and Supplementary Table 2). In further analysis of the differential expression of *T. laevis*-infected and control samples by using cluster expression analysis, the results showed that there were 0.8% more downregulated genes than upregulated genes in *T. foetida* plants (Figure 5).

### Gene Ontology Enrichment Analysis of Differentially Expressed Genes

**FIGURE 6:**
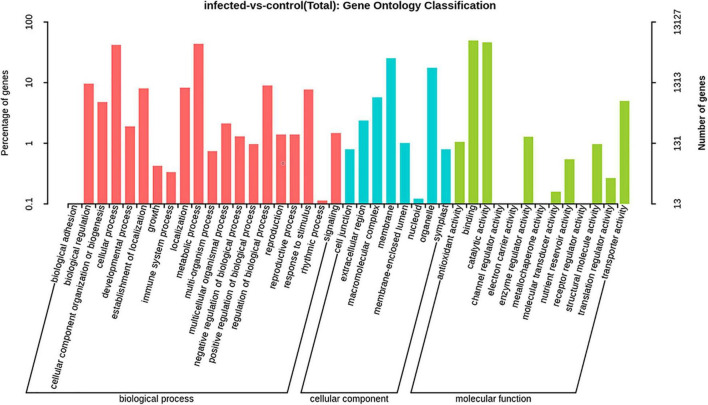
Gene ontology (GO) enrichment analysis of significant DEGs of *T. laevis*-infected and control samples. Annotations are grouped by biological process, cellular component, and molecular function.

**FIGURE 7:**
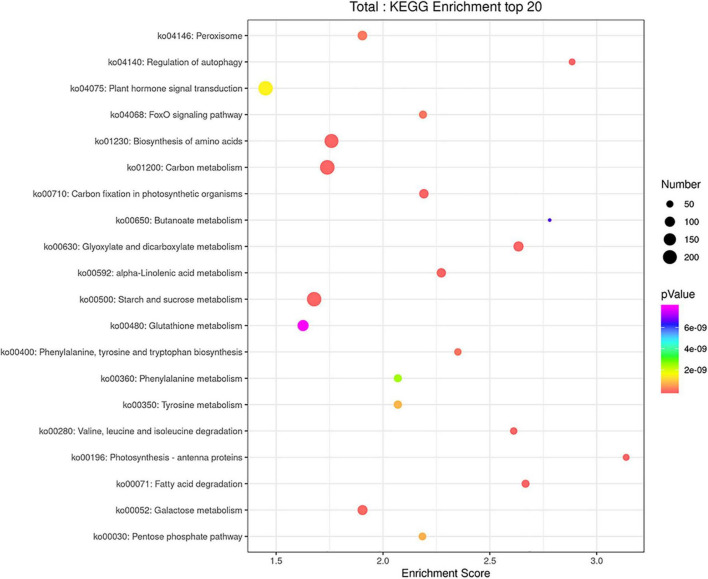
KEGG enrichment analysis scatter plot representing pathways of DEGs in response to *T. laevis* infection. The colors blue, white and red indicate low, medium, and high expression patterns of genes, respectively.

The GO categories were developed to evaluate potential DEG functions. The DEGs were classified into 48 functional categories, including biological process (21), cellular component (13), and molecular function (14). The results showed that in the biological process category, GO was associated mainly with cellular component organization, establishment of localization, localization, metabolic process, regulation of biological process, response to stimulus, and single organism process. Meanwhile, in the cellular component category, DEGs were primarily associated with cells, cell parts, membranes, membrane parts, and organelles. Similarly, in the molecular function category, DEGs were associated primarily with binding, catalytic activity, nucleic acid binding transcription factor activity, and transporter activity (Figure 6). In addition, KEGG pathway mapping was also carried out, and the results showed that 200 different pathways were identified (Supplementary Table 3). Of these pathways, 20 were peroxisome, regulation of autophagy, plant hormone signal transduction, FoxO signaling pathway, etc. (Figure 7).

### Differential Expression of Defense-Related Myeloblastosis Transcription Factor After *Tilletia laevis* Infection

Our results showed that after *T. laevis* infection, the expression of pathogenesis-related (PR) genes changed. The results showed that 6 pathogenesis-related genes, 3 thaumatin-like protein, 3 chitinase, 86 peroxidase, and 15 glucanase genes, changed during *T. laevis* infection, and their expression is shown in Supplementary Table 4. The expression of MYB transcription factors was also changed after *T. laevis* infection. The results showed that 10 of 14 and 4 of 14 MYB transcription factors were upregulated and downregulated, respectively (Supplementary Table 5).

### Validation of RNA Sequencing Results by Quantitative Real-Time PCR

**FIGURE 8:**
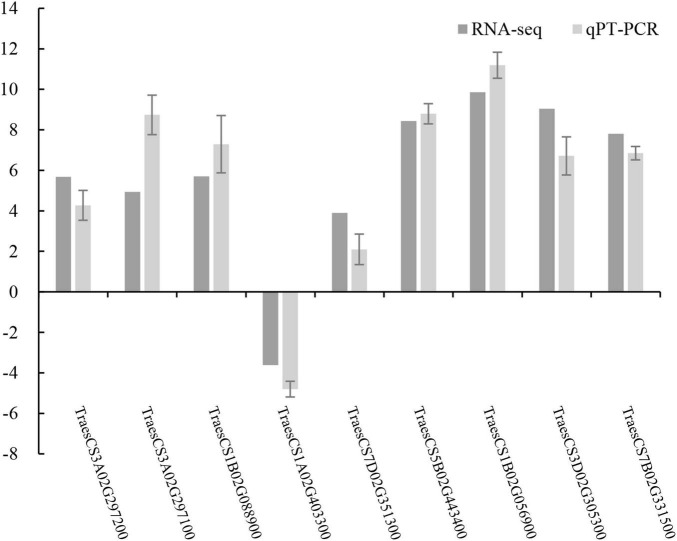
Validation of RNA-Seq data by quantitative real-time PCR (qRT-PCR).

To verify the reliability of the obtained transcriptome data, we screened out some of the differentially expressed genes for qRT-PCR verification. Taking the stress resistance of tassels infected by *T. laevis* as the screening standard, 9 differential genes were selected for quantitative verification. The expression trends in the qRT-PCR results validated the transcription sequencing results, which indicated a high degree of reproducibility between transcript abundances assayed using RNA-Seq and the expression profiles revealed by qRT-PCR data (Figure 8 and Supplementary Table 6).

## Discussion

Common bunt is one of the most serious diseases of wheat caused by *T. laevis*, and common grains are partly or totally replaced by fugus gulls, leading to 80% of wheat yield loss. To date, due to a lack of information on interactions between wheat tassels and *T. laevis* at the transcript level, therefore, it is essential to explore plant-pathogen interactions in the reproductive organs (tassels) of wheat. To determine plant responses to abiotic and biotic stresses, a large-scale gene expression analysis was performed (Desmond et al., 2008). Gene expression levels upon infection by pathogens have been broadly investigated in various crops using RNA-Seq, such as in wheat crops against *T. controversa* (Ren et al., 2020), *F. graminearum* (Erayman et al., 2015) and obligate pathogens (Poretti et al., 2021). In this study, based on RNA-Seq, we used the highly susceptible cultivar “Dongxuan 3” with *T. laevis* to analyze the response of the gene expression levels in fungal infected plants. Our findings showed that a different set of defense genes was differentially and specifically expressed and that a series of signaling molecules in wheat tassels were triggered by *T. laevis*.

Pathogenesis-related genes have key roles in the plant defense system against fungal pathogen infection (Sels et al., 2008). Based on the literature, thaumatin, chitinase, peroxidase, glucanase, and PR-10 enhance resistance to *T. controversa* and *F. pseudograminearum* in wheat (Desmond et al., 2008; Muhae-Ud-Din et al., 2020b; Chen et al., 2021), and *Ustilaginoidea virens* in rice (Han et al., 2015). Consistent with our findings, PR genes in wheat have been induced by *T. controversa* (Ren et al., 2020). We compared the transcript levels between *T. laevis*-inoculated and control plants in the tassels. The expression of 113 defense-related genes was changed after *T. laevis* inoculation (Supplementary Table 5), including 6 PRs, 3 thaumatin, 3 chitinase, 86 peroxidase, and 15 glucanase. Myeloblastosis (MYB) transcription factors form one of the largest protein superfamilies in plants and are involved in diverse biological processes, including cell wall biosynthesis, cell cycle regulation, reproduction and development, and play a significant role in controlling the transcription of defense-related genes (Millar and Gubler, 2005; Dubos et al., 2010; Zhang et al., 2015; Shan et al., 2016). For example, the R2R3-MYB transcription factor has increased the resistance in *Arabidopsis* against *Alternaria brassicicola* and *Botrytis cinerea* and was also involved in the tolerance of oxidative and osmotic stresses (Mengiste et al., 2003). The HvMYB6 improves the immunity level in barley against *Blumeria graminis* (Chang et al., 2013). Overexpression of the MYB gene TaPIMP1 induced a resistance in wheat against *Bipolaris sorokiniana* (Zhang et al., 2012). Our results were consistent with the above studies and showed that 14 MYB transcription factors were altered after *T. laevis* inoculation: 10 were upregulated and 4 were downregulated (Supplementary Table 5). The above changes in the transcription levels of different gene families might play a role in disease suppression against *T. laevis*.

Upon plant pathogen infection, plant defense responses contain transcriptional regulation of a large number of plant host genes. An RNA-Seq profiling suggests that plants activate different sets of defense-related genes to overcome severity of the disease in various crops (Conrath, 2011; Ren et al., 2020). Some defense-related proteins, such as thaumatin, PR proteins, chitinase, peroxidase, and glucanase, are capable of destroying the cell wall components of fungal pathogens and boosting the defense response of plants (Chen and Chen, 2002). In *Tilletia* species-infected wheat crops, the genes encoding chitinase, lipase, PR1.1, PR1.2, defensins and PR-10 were previously reported to be highly expressed in fungi-infected plants compared to control plants at different time intervals (Muhae-Ud-Din et al., 2020a). Desmond et al. (2008) reported that the expression of PR-1, β-1,3-glucanase, thaumatin-like proteins, and PR-10 was induced significantly in wheat crops after *F. pseudograminearum* infection. Similarly, the expression of peroxidase involved in reactive oxygen species (ROS) was induced after *F. pseudograminearum* infection (Kawano, 2003; Desmond et al., 2008). Transcription factors play roles in activating primary response genes after pathogen infection (Herschman, 1991; Li et al., 2016). Therefore, transcriptional regulation of plant genes is a part of the plant defense mechanism and plays an important role in inducing plant disease resistance (Chen and Chen, 2002). Similarly, in plant–pathogen interactions, pathogens take nutrients from plants, and plant cells try to stop nutrient movements by altering carbon metabolism and transport (Chen et al., 2011; Kretschmer et al., 2017; Kanwar and Jha, 2019). Some genes regulated the phytosynthetic activity repressed by affecting the photosynthetic activity (Rolfe and Scholes, 2010; Windram et al., 2012; Smith et al., 2014). In the present study, the transcription levels of 113 defense-related genes were changed after the *T. laevis* infection, including 6 PR genes, 3 thaumatin-like proteins, 3 chitinases, 86 peroxidases, and 15 glucanases (Supplementary Table 4). The 44 PRS genes were upregulated, and 69 PRs were downregulated. The above changes in the transcription levels of different gene families might play a role in the suppression of disease against *T. laevis*.

The GO enrichment analysis revealed that in the “biological process” category, cellular process, metabolic process, and single-organism process; in the “cellular component” category, cell, cell part, membrane, and membrane part; and in the “molecular function” category, binding and catalytic activity had the highest number of DEGs during plant pathogen interactions (Figure 6). These results indicated that the pathogens mobilized the primary and secondary metabolisms and finally regulated the expression of related genes through signal transduction and ion transport, which clearly induced the immune defense responses and may have a role in disease suppression. After pathogen infection, plants spend more energy in signaling and transportation processes to defend themselves than morph physiological and reproductive processes (Siemens et al., 2006; Berger et al., 2007). Furthermore, KEGG pathway analysis showed that most DEGs were characterized by biosynthesis of amino acids, carbon metabolism, and starch and sucrose metabolism (Figure 7). Amino acids have key roles in plant species, such as nitrogen sources, stress-reducing agents, hormone precursors (Zhao, 2010; Maeda and Dudareva, 2012) and as signaling factors of various physiological processes in plant species (Teixeira et al., 2018). Sucrose is an important element of assimilated carbon, which is a by-product during photosynthesis and then transported from source to sink tissues *via* the vascular system (Tauzin and Giardina, 2014). Overall, our findings showed that defense-related genes, including PR genes and MYB transcription, were mostly upregulated after *T. laevis* infection, suggesting that these gene families play important roles in common bunt resistance in wheat and may contribute to the control of wheat common bunt by regulating the over-expression defense genes.

## Data Availability Statement

The data presented in the study and deposited in the Supplementary Material.

## Author Contributions

LG designed the experiments. TH and ZR performed the experiments. GM and LG analyzed the data. TH, GM-U-D, and LG wrote the manuscript. WC, TL, and QG provided some materials. All authors reviewed the manuscript.

## Conflict of Interest

The authors declare that the research was conducted in the absence of any commercial or financial relationships that could be construed as a potential conflict of interest.

## Publisher’s Note

All claims expressed in this article are solely those of the authors and do not necessarily represent those of their affiliated organizations, or those of the publisher, the editors and the reviewers. Any product that may be evaluated in this article, or claim that may be made by its manufacturer, is not guaranteed or endorsed by the publisher.

## Footnotes

1
http://plants.ensembl.org/Triticum_aestivum/Info/Index

2
https://www.kegg.jp/kegg/kegg1.html

## Supplementary Material

Supplementary Figure 1 Morphological characterization of teliospores of *T. laevis.*

Supplementary Figure 2 Specific band and sequence of SCAR marker of *T. laevis*.
