# Supplementary material for: Transcriptomics Analysis of Wheat Tassel Response to Tilletia laevis Kühn, Which Causes Common Bunt of Wheat
Source: Front Plant Sci. 2022 Feb 22;13:823907. doi: 10.3389/fpls.2022.823907 (PMC8902468; doi:10.3389/fpls.2022.823907)
Supplement: Supplementary file 1 [file Table_1.doc]

| Sample | raw reads | clean reads | total mapped reads | uniquely mapped | Q30 | GC |
| --- | --- | --- | --- | --- | --- | --- |
| Control,1 | 49.69 M | 48.59 M | 42935380 (88.36%) | 36301512 (74.71%) | 93.75% | 53.35% |
| Control,2 | 50.29 M | 49.12 M | 44204310 (89.99%) | 37732069 (76.81%) | 93.96% | 52.74% |
| Control,3 | 48.64 M | 47.46 M | 42391424 (89.33%) | 35937004 (75.73%) | 93.58% | 52.74% |
| Infected,1 | 51.69 M | 50.69 M | 24042553 (47.43%) | 21629400 (42.67%) | 94.63% | 55.91% |
| Infected,2 | 48.75 M | 47.82 M | 21748000 (45.48%) | 19842072 (41.49%) | 94.79% | 56.20% |
| Infected,3 | 51.52 M | 50.30 M | 40159626 (79.85%) | 34459566 (68.51%) | 94.50% | 56.72% |

**Table S1.** Transcriptome analysis of RNA-Seq data. Infected-1 stands for *T. foetida* infected-1, Infected-2 stands for *T. foetida* infected-2, Infected-3 stands for *T. foetida* infected-3.
