# Supplementary material for: Transcriptomics Analysis of Wheat Tassel Response to Tilletia laevis Kühn, Which Causes Common Bunt of Wheat
Source: Front Plant Sci. 2022 Feb 22;13:823907. doi: 10.3389/fpls.2022.823907 (PMC8902468; doi:10.3389/fpls.2022.823907)
Supplement: Supplementary file 6 [file Table_6.docx]

Table S6. List of primers used for qRT-PCR

| gene_id | | log2Fold  Change | | pval | description | | Primer  (5'-3') |  |
| --- | --- | --- | --- | --- | --- | --- | --- | --- |
| TraesCS3A  02G297200 | 5.678340607 | | 0.00000125 | | Peroxidase 2 | F: GCGACCGCAAGATCAACATC  R: CCGTGAGGTTAGAGAAGGGC | | |
| TraesCS3A  02G297100 | 4.937036396 | | 0.002107936 | | Peroxidase 2 | F: GGGCCCTTCTCCAACCTTAC  R: TTGTTGTCGTTGCTGCACTG | | |
| TraesCS1B  02G088900 | 5.699828126 | | 1.61E-10 | | WRKY24 | F: AATGGCGCTGAGCCTGAGA  R: CTTGACAACCTTCTGCCCGT | | |
| TraesCS1A  02G403300 | -3.62033 | | 0.000165376 | | WRKY24 | F: GGCCGTTCTTGATCTCCTT  R: CAGAGCATCCTTGCCATCTG | | |
| TraesCS7D  02G351300 | 3.894389743 | | 0.009963031 | | Chitinase 1 | F: CTACACGTACGACGCCTTCA  R: GACGTGGCCTTGCTTATCTC | | |
| TraesCS3D  02G227400 | 6.851585938 | | 0.0000123 | | WRKY22 | F: CAAATGGCCGACGATTGGGATCTC  R: CTAGTCCCCCGCGAATCATA | | |
| TraesCS5B  02G288600 | -7.274222476 | | 4.89794E-08 | | bidirectional sugar transporter SWEET11-like | F: CCGAGTACATGCCCTTCTCG  R: TTCGGGAGGGTGACGTAGAT  F: TGGTGATCAAGACCAAGAGCG  R: CCTTGGTGAAGAGGCCGTAG  F: CTAACGATTTGCGAACCGACC  R: GGGCACGTGGAATGGTAGAT | | |
| TraesCS5B  02G443400 | 8.431467908 | | 0.005675397 | | Pathogenesis-related protein 1-7 | F: CCAAAACTCGGAGCAGGACT  R: CCGTAGAGGTTCTCCCCGTA  F: GTGCTGCTCGTAGCTCTCAT  R: ACCGTAGCATCCCATGTCAC  F: TGACATGGGATGCTACGGTG  R: GTACTGCTTCTCCGACACCC | | |
| TraesCS1B  02G056900 | 9.851180483 | | 1.77202E-10 | | Laccase | F: ATTCAGCAAGACTGTGGCGA  R: CGGTGAGGTTGGAGAGGAAC  F: TACACCACGCCGTACAAGAC  R: TCGCCACAGTCTTGCTGAAT  F: GCTACCACAACTGCCAGAG  R: GTGACAAACATGCGGGTGTC | | |
| TraesCS3D  02G305300 | 9.04624076 | | 1.14234E-07 | | Peroxidase | F: TCTCTGAGATGGACTCGGCA  R: TCTTGTCGACCACGTTCTGG  F: CCAGAACGTGGTCGACAAGA  R: CTTGACCCCGATTTTCCCCA  F: AAGAACAGCGACACGGAGAG  R: ATCTTGCGGTCGCTGAGAAA | | |
| TraesCS7B  02G331500 | 7.804191258 | | 2.19567E-16 | | Auxin efflux carrier component | F: GATGCAGTTCTGCCTCTGGT  R: CTTGGTGGACTCGCTCAACT  F: GCCTCGTCTGGGCTCTAATC  R: GCCATGAACGTCCCTGATGA  F: CTAGCTTCCTTGGCCTCGTC  R: GCCGCAAGGAATGAACTGTG | | |
