# Supplementary figures and images for: Transcriptomics Analysis of Wheat Tassel Response to Tilletia laevis Kühn, Which Causes Common Bunt of Wheat
Source: Front Plant Sci. 2022 Feb 22;13:823907. doi: 10.3389/fpls.2022.823907 (PMC8902468; doi:10.3389/fpls.2022.823907)

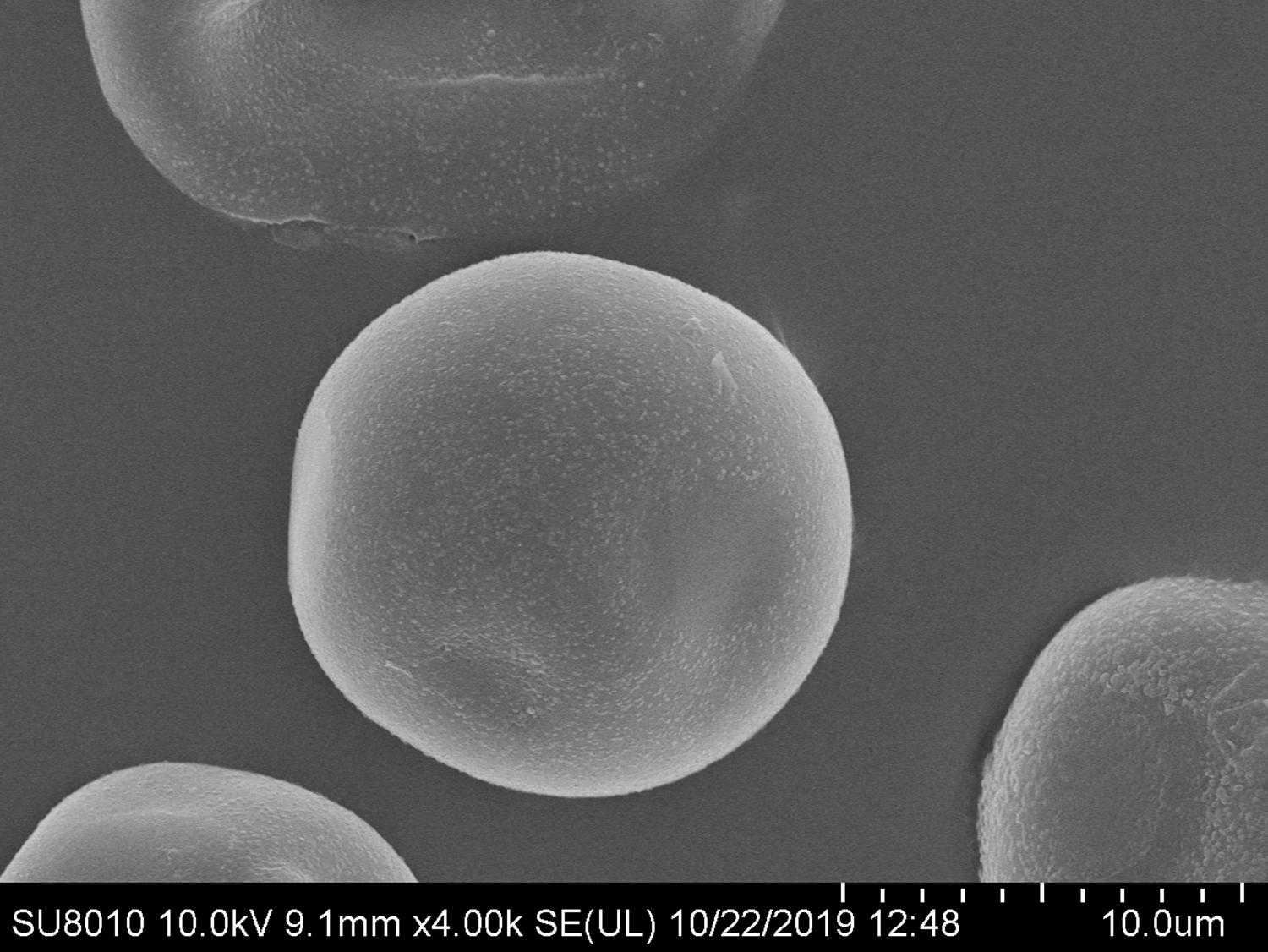

Supplement: Supplementary Figure 1 — Morphological characterization of teliospores of T. laevis. [file Image_1.tif]

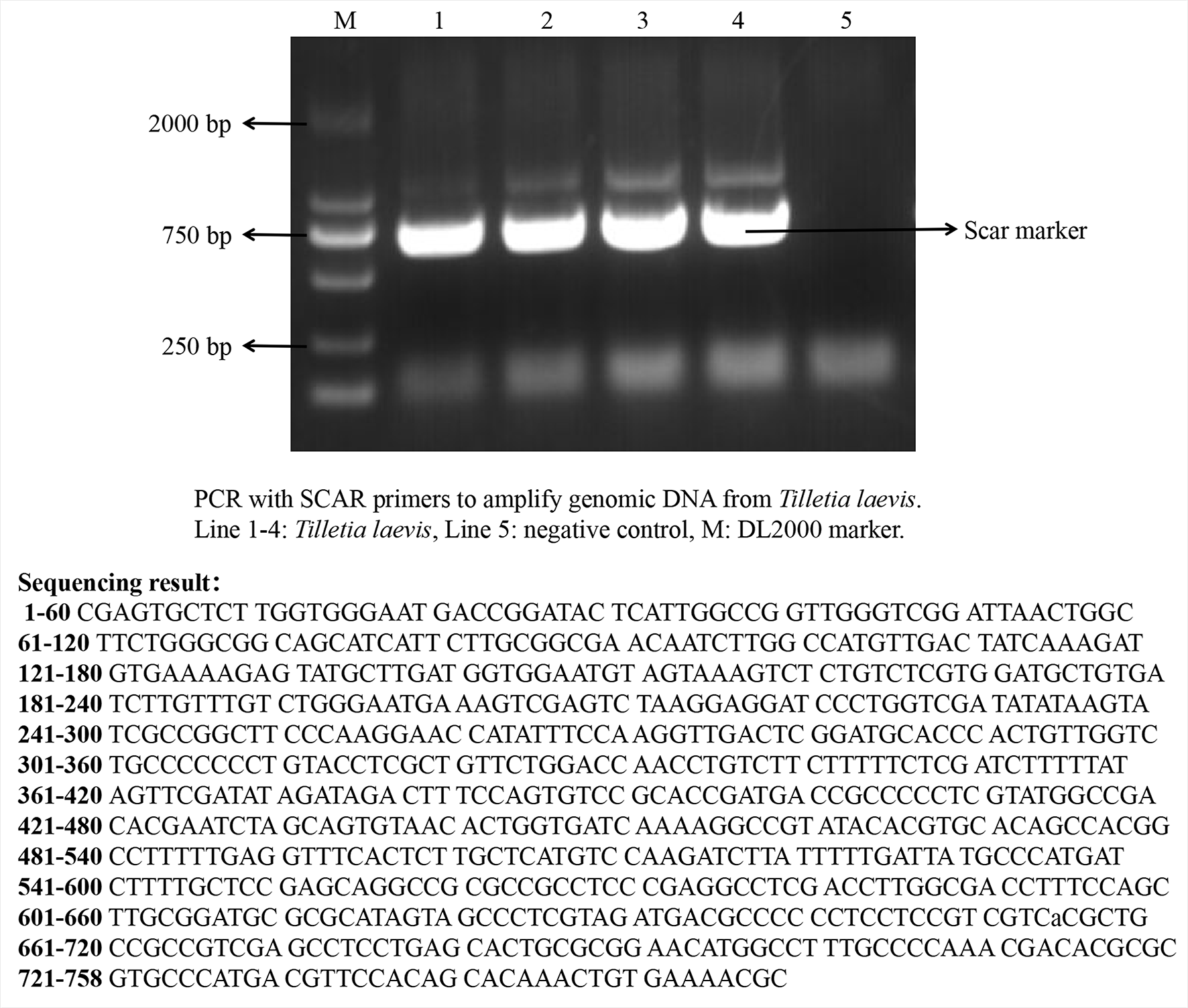

Supplement: Supplementary Figure 2 — Specific band and sequence of SCAR marker of T. laevis. [file Image_2.tif]
